# 

**Published:** 1901-07-06

**Authors:** 


### NOTICE TO CORRESPONDENTS.

All MSS., Letters, Books for Review, and other matters intended for the Editor should be addressed The Editor, "The Hospital," The Hospital Building, 28 & 29 Southampton Street, Strand, W.C.

The Editor cannot undertake to return rejected MS., even when accompanied by stamped directed envelope.

**Notice to Advertisers and Subscribers.**

All Advertisements, Orders for Copies of The Hospital, and Business Communications should be addressed to **THE MANAGER (Not to the Editor),** The Hospital, 28 & 29 Southampton Street, Strand, London, W.C.

The Cost of Subscription, post free, is as follows:—Per week, 2½d.; per quarter, 2s. 8d.; per half-year, 6s. 3d.; per annum, 10s. 6d. Foreign:—Per annum, 16s. 2d., payable, in all cases, in advance.

**NOTICE.—Vol. XXIX. of "The Hospital" (October 1900, to March, 1901), in handsome cloth binding is now on sale at the Office of this Journal, price, 7s. 6d. Cases for binding the Numbers contained in this Volume 1s. 6d. Index to Vol. XXIX., 2½d., post free.**

**The Monthly Part for July is now ready. Price 6d., by post 8d.**

### THE STUDENT OF MEDICINE.

### APPOINTMENTS.

**Joseph Anderson**, M.B., C.M.Aberd., appointed Honorary Surgeon to the Preston and County of Lancaster Royal Infirmary. **Harold Austen**, M.D., B.S.Lond., M.R.C.S., L.D.S.Eng., appointed Assistant Dental Surgeon to the Dental Hospital of London. **D. Cannan**, M.R.C.S., L.R.C.P.Lond., appointed District Medical Officer of the West Ham Union. **Sydney Clark**, M.R.C.S.Eng., L.R.C.P.Lond., appointed Honorary Visiting Medical Officer to the Victoria Home for Invalid Ladies, Leeds. **J. Craig**, L.R.C.P., L.R.C.S.Edin., appointed District Medical Officer of the Wearsdale Union. **E. L. Cropp**, M.R.C.S., L.R.C.P.Lond., appointed District Medical officer of the Reading Union. **E. P. Edwards**, L.R.C.P.Edin., L.F.P.&S.Glasg., appointed District Medical Officer of the Holyhead Union. **H. E. Gough**, L.R.C.P.Lond., M.R.C.S.Eng., appointed District Medical Officer of the Northwich Union. **Charles A. Hill**, M.B., B.A.Cantab., D.P.H.Vict., appointed Senior Assistant Physician to the Liverpool Hospital for Consumption and Chest Diseases. **Harvey Hilliard**, M.R.C.S., L.R.C.P.Lond., appointed Assistant Instructor in Anæsthetics at the London Hospital Medical College. **T. E. Hincks**, M.B., B.S.Edin., appointed Medical Officer of Health for the Hay Urban District Council. **W. F. M. Jackson**, L.R.C.P.Edin., M.R.C.S., appointed Medical Officer of Health for the Borough of Smethwick. **E. Harries Jones**, M.B., C.M.Edin., M.R.C.S.Eng., L.R.C.P.Lond., appointed Honorary Ophthalmic Surgeon to Northampton General Infirmary. **G. E. H. Le Fanu**, M.D.Aberd., appointed Assistant Medical Officer in the Derby Borough Asylum. **Thomas Lumsden**, M.D.Aberd., Ch.B., appointed Registrar to Out-patients Central London Throat and Ear Hospital. **John Howson Ray**, M.B., Ch.M.Vict., F.R.C.S.Eng., appointed Assistant Surgeon to the Manchester Children's Hospital. **J. S. Rosser**, M.D.Glasg., D.P.H., F.P.S.Glasg., appointed Medical Officer of Health for the Llandovery Rural District. **A. W. Seal**, M.R.C.S.Eng., L.R.C.P.Lond., appointed First Assistant Medical Officer and Dispenser to the Central London Sick Asylum, Cleveland Street. **F. E. Streeten**, L.R.C.P.Edin., M.R.C.S.Eng., appointed Medical Officer of Health for the Borough of Swindon. **R. Deane Sweeting**, M.A.Oxon., M.D.Dub., D.P.H.Camb., Senior Medical Inspector of the Local Government Board, appointed Examiner in Public Health and Preventive Medicine in the University of Oxford.

28 & 29 SOUTHAMPTON STREET, STRAND, W.C.

